# Targeted Endogenous Bioelectric Modulation in Autism Spectrum Disorder: Real-World Clinical Outcomes of the REAC BWO Neurodevelopment–Autism Protocol

**DOI:** 10.3390/jcm14217500

**Published:** 2025-10-23

**Authors:** Arianna Rinaldi, Hingrid Angélica Benetti Mota, Salvatore Rinaldi, Vania Fontani

**Affiliations:** 1 Department of Adaptive Neuro Psycho Physio Pathology and Neuro Psycho Physical Optimization, Rinaldi Fontani Institute, 50144 Florence, Italy; ari@irf.it (A.R.);; 2 Department of Regenerative Medicine, Rinaldi Fontani Institute, 50144 Florence, Italy; 3 Research Department, Rinaldi Fontani Foundation, 50144 Florence, Italy; 4 International Scientific Society of Neuro Psycho Physical Optimization with REAC Technology-Brazilian Branch, São Paulo 01126-000, Brazil

**Keywords:** Autism Spectrum Disorder, neuromodulation, Radio Electric Asymmetric Conveyer, Brain Wave Optimization, endogenous bioelectric activity, sensory integration

## Abstract

**Background:** Autism Spectrum Disorder (ASD) is characterized by atypical brain oscillatory dynamics and altered connectivity, impairing sensory integration, socio-communicative responsiveness, and behavioral regulation. **Methods**: Radio Electric Asymmetric Conveyer (REAC) technology delivers non-invasive neurobiological modulation through standardized, operator-independent protocols. The Brain Wave Optimization Neurodevelopment–Autism (BWO ND-A) protocol was designed to address oscillatory patterns frequently altered in ASD, aiming to promote network coherence and multidomain functional improvement. This retrospective pre–post single-arm study evaluated 39 children with ASD (31 males, 8 females; mean age 7.85 ± 2.90 years). All received one Neuro Postural Optimization (NPO) session to prime central nervous system adaptive capacity, followed by BWO ND-A (18 sessions, ~8 min each), administered 3–4 times daily over ~two weeks. The primary outcome was the Autism Treatment Evaluation Checklist (ATEC) total score; secondary outcomes were its four subscales. **Results:** Mean total ATEC decreased from 67.76 ± 16.11 to 56.25 ± 23.66 (mean change −11.51 ± 14.48; *p* < 0.0001; Cohen’s dz = 0.78). Clinically meaningful improvement (≥8-point reduction) occurred in 59% of participants. In 10.3% of cases, caregiver ratings indicated an apparent worsening (≥8-point increase). However, no objective deterioration or adverse effects were observed. This pattern was most likely related to a transient phase of functional re-adaptation, during which emerging changes may initially be perceived by caregivers as worsening before stabilizing into improvement. **Conclusions:** While these findings suggest promising short-term real-world efficacy and safety, the absence of a control group, lack of objective neurophysiological measures, and no long-term follow-up limit causal inference. Future controlled studies with neurophysiological monitoring are needed to confirm the targeted neuromodulatory action and durability of effects.

## 1. Introduction

Autism Spectrum Disorder (ASD) is a heterogeneous neurodevelopmental condition defined by persistent deficits in social communication and interaction, alongside restricted interests and repetitive behaviors [1]. Affecting approximately 1–2% of the global population [2,3], ASD imposes a substantial individual, familial, and societal burden [4]. Beyond its core diagnostic features, many individuals exhibit sensory processing abnormalities [5], impaired cognitive flexibility [6], and difficulties in emotional and behavioral regulation [7]. These associated features often exacerbate functional impairment and reduce participation in educational, social, and community life.

The neurobiological underpinnings of ASD are multifaceted [8], involving genetic, epigenetic, and environmental factors that converge on atypical brain development [9]. Converging evidence from neuroimaging and electrophysiological studies has highlighted the role of aberrant neural oscillations [10] and altered functional connectivity across large-scale networks [11]. Such dysregulation affects the temporal coordination of neural activity both within and between cortical and subcortical structures [12,13], thereby compromising the neural basis of perception, cognition, socio-emotional behaviors, and adaptive functioning [14]. Specifically, dysregulated oscillatory activity has been linked to impaired sensory integration [15,16,17], diminished socio-communicative responsiveness [14], and increased behavioral rigidity domains that are often resistant to conventional therapeutic strategies [15,18].

Current treatment paradigms for ASD primarily rely on behavioral and educational interventions [19], sometimes supplemented by symptom-targeted pharmacotherapy [20]. While these approaches can yield measurable improvements, their impact is often partial and they do not directly address the neurophysiological mechanisms of the disorder. This has spurred interest in novel interventions aimed at modulating brain network dynamics, with the goal of promoting adaptive plasticity and improving functional outcomes. Among these, non-invasive neurobiological modulation techniques have emerged as promising adjunctive options, capable of influencing endogenous neural activity without the risks associated with invasive procedures [21,22]. However, most existing non-invasive neuromodulation methods require individualized parameter adjustment, may show variable reproducibility, and are not always feasible for intensive treatment cycles in pediatric populations.

Radio Electric Asymmetric Conveyer (REAC) technology was developed to interact with the body’s endogenous bioelectric activity in a targeted and asymmetrically conveyed manner, promoting functional optimization of neural circuits [23,24].

Its therapeutic protocols are pre-set and operator-independent. Previous experimental and clinical studies have demonstrated that REAC technology exerts its effects selectively in tissues and neural circuits where alterations of endogenous bioelectrical activity are present, without interfering with healthy structures [23,24,25,26,27]. Previous REAC applications in neurodevelopmental and neuropsychiatric contexts particularly through Neuro Postural Optimization (NPO), Neuro Psycho Physical Optimization (NPPO), and Neuro Psycho Physical Optimization–Cervico-Brachial (NPPO-CB) protocols have demonstrated improvements in cognitive performance [28], behavioral adaptation, and emotional regulation [29,30,31,32]. In ASD populations, these protocols have been associated with enhanced adaptive functioning and reduced behavioral dysregulation [21,33,34].

Building on this foundation, the REAC brain wave optimization (BWO) Neurodevelopment–Autism (BWO ND-A) protocol was specifically conceived to address electrophysiological patterns frequently altered in ASD. It employs a standardized sequence of endogenous modulation steps targeting neural oscillations implicated in sensory integration, socio-emotional processing, and attentional control. By restoring network coherence and improving cross-frequency coupling, BWO ND-A seeks to enhance information processing and promote adaptive behavioral and cognitive responses.

Given its specific neurofunctional targeting and the encouraging evidence from previous REAC applications in ASD [21,33,34], the BWO ND-A protocol preceded by a single NPO session to prime central nervous system modulatory capacity [23] represents a promising therapeutic option with potential impact beyond the clinical setting. By addressing core and associated symptoms through standardized, reproducible neurobiological modulation, this approach may offer tangible benefits for individuals with ASD and their families, complementing existing behavioral and educational interventions. The present study provides real-world evidence of its multidomain efficacy, supporting its integration not only in clinical practice but also in broader social and community-based initiatives aimed at improving quality of life and social participation in ASD.

## 2. Materials and Methods

### 2.1. Overview of REAC Technology and BWO ND-A Protocol

REAC technology (Radio Electric Asymmetric Conveyer) neurobiological modulation platform designed to interact with endogenous bioelectric activity [23,24,25]. Treatments are delivered through Asymmetric Conveyor Probes (ACPs), which asymmetrically convey very low-intensity radio electric fields to the body. All therapeutic protocols are pre-set, operator-independent, and standardized to ensure reproducibility and safety. Unlike other neuromodulation techniques, REAC acts selectively on sites with altered endogenous bioelectrical activity [35], without affecting areas functioning within normal parameters.

The BWO ND-A protocol is specifically configured to modulate brain oscillatory patterns frequently altered in ASD [36,37], with the aim of improving sensory integration [15,16,17], socio-communicative responsiveness, and behavioral regulation [3]. It consists of a standardized and non-modifiable sequence of modulation steps, developed from prior clinical and neurophysiological research [37,38,39], and is delivered identically across all patients to ensure comparability of outcomes.

### 2.2. Relation to Other REAC Neurobiological Modulation Protocols

Within the REAC neurobiological modulation platform, Brain Wave Optimization (BWO) protocols are preconfigured to modulate specific patterns of brain oscillatory activity. Standard or “base” BWO protocols target a single specific frequency within a fundamental brain rhythm, such as BWO-Delta (deep sleep rhythms), BWO-Theta (drowsiness and meditative states), BWO-Alpha (relaxation and quiet wakefulness), and BWO-Gamma (complex cognitive processing). Each of these base protocols acts on functions that are highly specific to the selected frequency.

Over time, clinical and neurophysiological research revealed that many functional alterations involve not only individual frequency bands but also their dynamic cross-frequency orchestration [37,38,39]. This led to the development of “composite” BWO protocols, each consisting of a specific sequence of modulations drawn from the base protocols, tailored to specific clinical profiles.

The Neuro Psycho Physical Optimization—Cervico-Brachial (NPPO-CB) was the first clinically introduced BWO protocol, historically referred to without the BWO suffix for ease of communication [40]. It primarily operates within the delta frequency range and is aimed at subcortical neurobiological modulation for emotional adaptation.

From this experience, further composite protocols were developed. The NPPO-CB BWO Neurodevelopment—Autism (BWO ND-A) used in this study combines modulations from multiple base frequency bands to re-orchestrate altered brain rhythms frequently observed in ASD, targeting sensory over/under-responsiveness, socio-communicative impairments, and behavioral inflexibility.

### 2.3. Study Design and Setting

This retrospective observational pre–post clinical study was conducted at a single clinical center under the coordination of the Rinaldi Fontani Institute & Foundation (Florence, Italy), with treatments delivered in accordance with the same standardized clinical protocols and data collection procedures established by the coordinating institute. The aim was to assess changes in global and domain-specific symptomatology in individuals with ASD following the REAC BWO Neurodevelopment—Autism (BWO ND-A) protocol, preceded by a single NPO session. The retrospective design was chosen to capture real-world data from routine clinical practice, allowing the inclusion of all consecutive eligible cases over the observation period. All procedures were performed according to established clinical practice, by experienced personnel trained in REAC methodology.

### 2.4. Participants

Participants were 39 children with a prior clinical diagnosis of ASD, confirmed by specialist evaluation. This cohort represents the entire population of ASD patients treated in the first seven months of the year at participating center, with no prior history of REAC treatments. Inclusion criteria were: (1) age between 4 and 13 years, (2) confirmed ASD diagnosis, (3) ability to complete the planned treatment cycle, and (4) stable pharmacological and behavioral treatment regimens for at least eight weeks prior to study entry. Exclusion criteria included: (1) acute medical conditions, (2) recent changes in ongoing therapies, and (3) inability to comply with the treatment schedule.

Of the 39 participants, 31 (79.5%) were male and 8 (20.5%) were female, with a mean age of 7.85 ± 2.90 years (range 4–13). Age distribution was as follows: 14 participants (35.9%) were aged 4–6 years, 13 (33.3%) were 7–9 years old, and 12 (30.8%) were 10–13 years old. All completed the treatment cycle without adverse events (Table 1).

Baseline demographic and clinical data were collected from medical records and verified by the treating clinicians.

All participants continued their pre-existing pharmacological and behavioral treatments unchanged during the REAC intervention. Only children whose therapeutic regimens had been stable for at least eight weeks prior to enrollment were included, to minimize confounding from recent treatment adjustments.

### 2.5. Intervention

All participants first received a single session of the NPO protocol, lasting only a few milliseconds, designed to optimize central nervous system adaptive capacity and prime the response to subsequent REAC treatments [23,41].

Following NPO, each underwent the BWO ND-A protocol: 18 sessions of ~8 min each, over one to two weeks, delivered 3–4 times per day with at least one hour between sessions (max four per day), according to established REAC safety parameters.

Treatments used a REAC medical device (BENE mod 110, ASMED, Scandicci, Italy) with asymmetric conveyer probe (ACPs) positioned in the cervico-brachial area per standard procedure. The ACP does not deliver current but channels interaction between the device’s radio electric field and endogenous bioelectrical activity. Protocol parameters are fixed and cannot be modified by the operator.

All treatment sessions were conducted in a dedicated clinical center under the supervision of personnel trained in REAC methodology. Post-intervention ATEC evaluations were conducted within one week after the completion of the treatment cycle.

### 2.6. Outcome Measures

The primary outcome was the change in ATEC total score from pre- to post-intervention [42]. Secondary outcomes were changes in its four subscales: (1) Speech/Language/Communication, (2) Sociability, (3) Sensory/Cognitive Awareness, and (4) Health/Physical/Behavior [43]. Assessments occurred within one week before and after the treatment cycle. The ATEC, completed by parents or primary caregivers, has been validated for ASD research [43,44] and used in prior REAC studies [34].

### 2.7. Statistical Analysis

Analyses used SPSS v.22 (IBM Corp., Armonk, NY, USA). Continuous variables are mean ± SD. Pre–post differences were tested with paired-sample *t*-tests (*p* < 0.05, two-tailed). Effect sizes were calculated as Cohen’s dz with 95% CI. which represents the standardized mean difference between pre- and post-treatment measures within the same participants, with 95% confidence intervals (CI) indicating the precision of the estimated effect sizes. No imputation for missing data was necessary, as all participants completed the study.

### 2.8. Ethical Considerations

The study was approved by the Institutional Review Board of the Rinaldi Fontani Institute (Protocol: IRB-RFI-2025-07-1-2). Given its retrospective nature and use of standard clinical practice, additional local IRB approvals were not required. All data were anonymized, and written informed consent for research use was obtained from parents or legal guardians. All procedures were conducted in accordance with the Declaration of Helsinki.

## 3. Results

All thirty-nine participants completed the treatment cycle, and no adverse events were reported during the study period. Treatment adherence was 100%, with no interruptions or dropouts, and all sessions were performed according to the standardized protocol.

**Figure 1:**
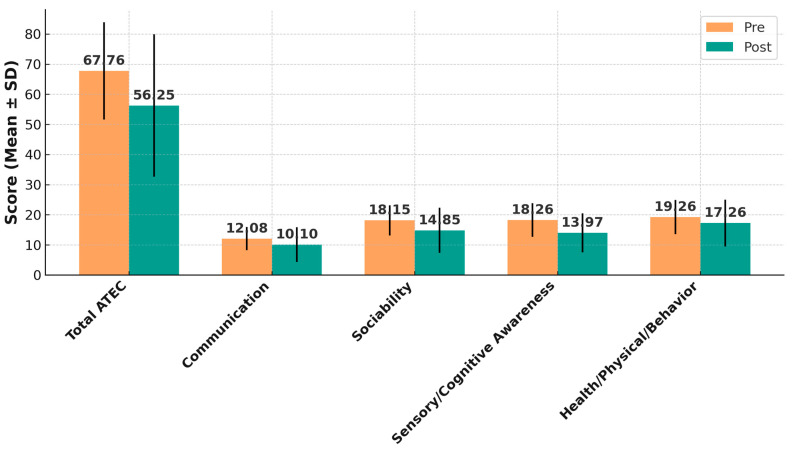
Pre- and post-treatment mean ATEC total and subscale scores with standard deviations. Bar chart illustrating the mean ± SD for the total Autism Treatment Evaluation Checklist (ATEC) score and each subscale Communication, Sociability, Sensory/Cognitive Awareness, and Health/Physical/Behavior before (Pre) and after (Post) the REAC BWO ND-A intervention. All domains demonstrated significant reductions in scores following treatment. Lower ATEC scores indicate symptom improvement across all domains.

At baseline, the mean total ATEC score for the cohort was 67.76 ± 16.11. Following the intervention, the mean total score decreased to 56.25 ± 23.66, corresponding to a mean reduction of 11.51 ± 14.48 points (95% CI: 6.98 to 16.03; t(38) = 4.90; *p* < 0.0001). This change represents a large treatment effect, as indicated by a Cohen’s dz value of 0.78 (95% CI: 0.43 to 1.11) (Table 2 and Figure 1).

Clinically significant improvements, defined as a reduction of at least eight points in the total ATEC score, were observed in 23 participants (59.0%; 95% CI: 42.1–74.4%) (Table 3). No clinically relevant change (<8 points) was recorded in 12 participants (30.8%) (Table 3). In 10.3% of participants, caregiver ratings suggested an apparent worsening, primarily in the Health/Physical/Behavior domain (Table 3). No objective deterioration was observed, and no adverse effects occurred. This phenomenon was interpreted as a transient disorientation phase in the process of functional reorganization, rather than a true negative outcome.

Improvements were evident across all four ATEC subscales (Table 2 and Figure 1). In the Communication domain, mean scores decreased from 12.08 ± 3.86 to 10.10 ± 5.74 (mean change 1.97 ± 5.10; 95% CI: 0.33 to 3.60; *p* = 0.021; dz = 0.38, 95% CI: 0.06–0.69). In Sociability, baseline scores of 18.15 ± 5.01 declined to 14.85 ± 7.51 (mean change 3.31 ± 6.48; 95% CI: 1.19 to 5.43; *p* = 0.0032; dz = 0.50, 95% CI: 0.17–0.82). Sensory/Cognitive Awareness scores demonstrated the largest improvement, dropping from 18.26 ± 5.56 to 13.97 ± 6.48 (mean change 4.28 ± 6.23; 95% CI: 2.25 to 6.31; *p* = 0.00014; dz = 0.68, 95% CI: 0.33–1.02). In the Health/Physical/Behavior domain, mean scores fell from 19.26 ± 5.70 to 17.26 ± 7.75 (mean change 2.00 ± 5.71; 95% CI: 0.14 to 3.86; *p* = 0.036; dz = 0.35, 95% CI: 0.03–0.66).

### 3.1. Outcomes by Age Group

Participants aged 4–6 years (n = 14) had the largest mean ATEC reduction (−13.4 points), followed by ages 7–9 years (−11.1) and 10–13 years (−9.6). Although differences between groups were not statistically significant, the trend suggests potentially greater responsiveness in younger children.

### 3.2. Outcomes by Sex

**Figure 2:**
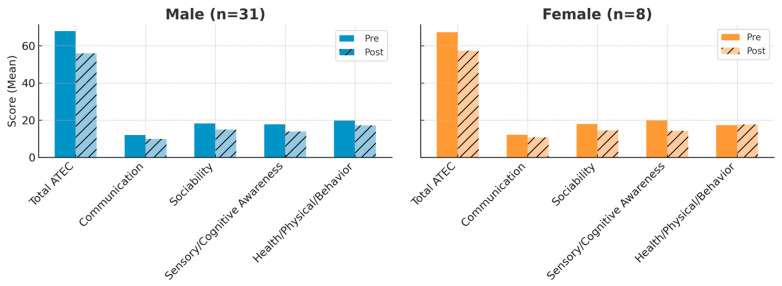
Pre- and post-intervention mean ATEC total and subscale scores by sex. The left panel shows male participants (n = 31) and the right panel shows female participants (n = 8). For each sex, mean scores are displayed for the total Autism Treatment Evaluation Checklist (ATEC) score and each subscale (Communication, Sociability, Sensory/Cognitive Awareness, and Health/Physical/Behavior) before (orange solid bars) and after (orange hatched bars for females; blue solid bars and blue hatched bars for males) the REAC BWO ND-A intervention. Both sexes demonstrated improvements across all outcome measures, with females showing slightly larger relative gains in Sensory/Cognitive Awareness and males exhibiting marginally greater improvements in Sociability. Lower ATEC scores indicate symptom improvement.

Both sexes improved in all outcome measures. In males, the mean total ATEC score decreased from 67.87 to 55.96; in females, from 65.21 to 54.91. Females showed slightly greater relative gains in Sensory/Cognitive Awareness, while males had marginally larger improvements in Sociability (Figure 2).

### 3.3. Clinical Observations

Caregivers frequently reported qualitative improvements consistent with the measured outcomes, including greater social engagement, improved responsiveness to verbal cues, and reduced behavioral rigidity. Additional comments described better tolerance of sensory stimuli, fewer tantrums, and more consistent eye contact. Although not formally quantified, these observations support the multidomain benefits detected by standardized assessments.

## 4. Discussion

This study provides real-world clinical evidence supporting the efficacy of the REAC BWO Neurodevelopment—Autism (BWO ND-A) protocol, preceded by a single Neuro Postural Optimization (NPO) session, in improving core and associated symptoms of ASD. The observed reduction in total ATEC scores [42,43], with a large effect size (Cohen’s dz = 0.78, 95% CI: 0.43–1.11) [45] and a clinically significant improvement in 59% of participants, suggests a relevant impact across multiple functional domains. These changes were achieved in a heterogeneous pediatric cohort aged 4–13 years, with a male-to-female ratio consistent with epidemiological trends in ASD prevalence [46]. The improvement pattern, with the largest gains in Sensory/Cognitive Awareness and Sociability, closely matches the neurofunctional targets of the BWO ND-A sequence, consistent with a specific rather than diffuse neuromodulatory effect.

From a neurophysiological standpoint, ASD is characterized by atypical oscillatory patterns [36,37] and disrupted connectivity in large-scale neural networks [11], affecting sensory integration, socio-communicative responsiveness, and cognitive flexibility [3]. The BWO ND-A protocol is specifically designed to target and modulate these electrophysiological patterns through a standardized, operator-independent sequence of endogenous bioelectric modulation steps. By enhancing coherence within frequency bands and restoring balanced cross-frequency coupling, the protocol may facilitate more efficient information flow between cortical and subcortical structures, thereby improving functional outcomes in targeted domains.

The most pronounced improvements in this study were observed in the Sensory/Cognitive Awareness and Sociability subscales, domains closely aligned with the neurofunctional targets of the protocol. Gains in Speech/Language/Communication and Health/Physical/Behavior, although of moderate effect size, further support the multidomain benefits of BWO ND-A. Qualitative reports from caregivers, such as increased social engagement, improved responsiveness to verbal cues, reduced behavioral rigidity, and better tolerance of sensory input, were consistent with quantitative findings and provide additional insight into the real-life impact of the intervention.

Previous studies on REAC neurobiological modulation in ASD, particularly those employing NPO and NPPO protocols, have demonstrated improvements in cognitive, behavioral, and emotional regulation domains [21,33,34]. In these earlier protocols, NPO was used to prime the central nervous system’s modulatory capacity before delivering broader neuromodulatory interventions [23,41]. The present study follows this same methodological approach but applies a more targeted protocol BWO ND-A, designed specifically to modulate cortical oscillatory sequences associated with ASD. This targeted approach may help explain why improvements were more concentrated in sensory [17] and social domains [38,47], while still extending to communication [48] and behavior regulation.

When compared to other non-invasive neuromodulation modalities, such as transcranial direct current stimulation (tDCS) [49] and repetitive transcranial magnetic stimulation (rTMS) [50], BWO ND-A offers several advantages: no direct current delivery, operator-independent administration, no need for individualized parameter adjustment, short sessions, and the possibility of multiple daily applications without increased risk. These features make it suitable for pediatric populations, including those less tolerant of conventional neuromodulation.

In addition, randomized controlled trials of other non-invasive brain stimulation approaches, including tDCS and rTMS, have also reported large effect sizes on ATEC scores, in some cases comparable or greater than those observed here (e.g., SMD ≈ 0.97 [49,51]). Our findings should therefore be interpreted as consistent with, rather than superior to, these emerging neuromodulation methods. The distinctive value of REAC BWO ND-A lies in its operator-independence, absence of current delivery, short and well-tolerated sessions, and feasibility of multiple daily applications in pediatric populations. These characteristics make it particularly adaptable to real-world clinical practice.

Against this backdrop, the effect size observed in our cohort (dz = 0.78) falls within the upper range of those reported in comparable interventions, particularly considering the short treatment cycle of one to two weeks. While the absence of a control group and objective biomarkers prevents definitive conclusions, these findings suggest that the BWO ND-A protocol may provide clinically meaningful improvements within a timeframe shorter than that usually reported for alternative interventions.

A minority of caregiver reports (10.3%) suggested apparent worsening. This was not accompanied by objective deterioration or adverse events and was most likely a temporary adaptive fluctuation, akin to transient reorganization phenomena documented in post-stroke recovery and neuromodulation contexts [52,53,54], rather than a genuine worsening.

The consistency of improvements across sexes and age groups supports generalizability, although trends toward greater benefit in younger participants suggest that early intervention may yield enhanced neuroplastic effects consistent with literature on critical developmental periods. This warrants confirmation in larger, stratified samples.

The single-group pre–post design without a control group limits causal inference, as placebo effects or regression to the mean cannot be entirely excluded. Nevertheless, several considerations reduce the likelihood that the observed changes were solely due to placebo. The magnitude of improvements, particularly in Sensory/Cognitive Awareness, is consistent with neurofunctional targets previously documented in REAC protocols [23,24,25,31,32] and is unlikely to be entirely attributable to expectancy alone. Moreover, the short duration of the intervention cycle (one to two weeks) makes it improbable that natural developmental progression could account for the observed effects. These findings are also in line with prior REAC studies conducted in independent cohorts and settings, where similar multidomain improvements were consistently observed [31,32]. Additionally, the multidomain structure of the ATEC reduces the risk that isolated subjective impressions could significantly influence the total score.

The sample size, while adequate for detecting large effects, remains modest, and the heterogeneity in age and baseline severity may have influenced responsiveness. The absence of objective neurophysiological measures, such as quantitative EEG, limits the mechanistic interpretation of the results, although previous REAC research has demonstrated protocol-specific reorganization of brain network activity [23,24]. Future research should therefore aim to replicate these findings in larger, multicenter observational cohorts, ideally with age- and severity-matched comparison groups to strengthen causal inference. Where possible, integrating non-invasive neurophysiological monitoring could provide direct evidence of oscillatory modulation and clarify the neural mechanisms underlying clinical improvements. Long-term follow-up would also be valuable to assess the persistence of benefits and the potential cumulative effects of repeated treatment cycles. Given the safety, tolerability, and reproducibility of the intervention, prospective randomized controlled trials would be both feasible and ethically justifiable.

## 5. Conclusions

In this real-world observational study, the REAC BWO Neurodevelopment–Autism (BWO ND-A) protocol, preceded by a single NPO session, produced significant multidomain improvements in children with ASD, with a large overall effect size and clinically meaningful changes in the majority of participants. The most notable gains were in sensory/cognitive awareness and sociability domains, which are closely linked to the specific neurofunctional targets of the protocol and suggest a targeted neuromodulatory action rather than a non-specific improvement. Additional benefits were observed in communication and behavioral regulation, further confirming the multidomain relevance of the intervention.

These findings, which are consistent with previous REAC neurobiological modulation studies, strengthen the rationale for integrating BWO ND-A as a safe, non-invasive, and operator-independent adjunct to established therapeutic programs for ASD. The standardization of the protocol ensures reproducibility across different settings, making it adaptable not only to specialized clinical environments but also to broader community and educational contexts where early and intensive interventions are beneficial.

Given the absence of reported adverse events, the short treatment duration, and the possibility of multiple daily sessions, BWO ND-A emerges as a feasible option for large-scale implementation in ASD care pathways. While further controlled prospective studies are necessary to confirm these results and clarify long-term outcomes, the present evidence supports the inclusion of this approach in multidisciplinary management strategies aimed at improving adaptive functioning, social participation, and quality of life for individuals with ASD and their families.

## Figures and Tables

**Table 1:** Distribution of participants by sex and age group. Age groups are expressed in years. Percentages are calculated relative to the total sample size (*n* = 39).

Characteristic	N (%)/Mean ± SD
Male	31 (77.5%)
Female	8 (22.5%)
Age (years)	7.85 ± 2.90 (range 4–13)

**Table 2:** Pre- and post-intervention ATEC total and subscale scores (n = 39). Data are expressed as mean ± standard deviation (SD). Mean change refers to the difference between pre- and post-treatment values. Effect sizes are reported as Cohen’s dz with 95% confidence intervals.

Domain	Pre-Treatment Mean ± SD	Post-Treatment Mean ± SD	Mean Change ± SD	95% CI Change	t (df = 38)	*p*-Value	Cohen’s dz (95% CI)
Total ATEC	67.76 ± 16.11	56.25 ± 23.66	11.51 ± 14.48	6.98 to 16.03	4.90	<0.0001	0.78 (0.43–1.11)
Communication	12.08 ± 3.86	10.10 ± 5.74	1.97 ± 5.10	0.33 to 3.60	2.40	0.021	0.38 (0.06–0.69)
Sociability	18.15 ± 5.01	14.85 ± 7.51	3.31 ± 6.48	1.19 to 5.43	3.15	0.0032	0.50 (0.17–0.82)
Sensory/Cognitive Awareness	18.26 ± 5.56	13.97 ± 6.48	4.28 ± 6.23	2.25 to 6.31	4.24	0.00014	0.68 (0.33–1.02)
Health/Physical/Behavior	19.26 ± 5.70	17.26 ± 7.75	2.00 ± 5.71	0.14 to 3.86	2.17	0.036	0.35 (0.03–0.66)

Abbreviations: ATEC, Autism Treatment Evaluation Checklist; SD, standard deviation; CI, confidence interval; df, degrees of freedom.

**Table 3:** Distribution of individual changes in total ATEC scores following the REAC BWO ND-A intervention.

Category	*n*	%	95% CI
Clinically significant improvement (≥8 points)	23	59.0	42.1–74.4
No clinically relevant change (<8 points)	12	30.8	17.0–47.6
Clinically significant worsening (≥8 points increase)	4	10.3	3.3–25.0

## Data Availability

All data supporting the findings of this study are contained within the manuscript.
